# A comparison of cost and quality of three methods for estimating density for wild pig (*Sus scrofa*)

**DOI:** 10.1038/s41598-020-58937-0

**Published:** 2020-02-06

**Authors:** Amy J. Davis, David A. Keiter, Elizabeth M. Kierepka, Chris Slootmaker, Antoinette J. Piaggio, James C. Beasley, Kim M. Pepin

**Affiliations:** 1 United States Department of Agriculture, Animal and Plant Health Inspection Service, Wildlife Services, National Wildlife Research Center, 4101 Laporte Avenue, Fort Collins, CO 80521 USA; 2 University of Georgia, Savannah River Ecology Laboratory, D. B. Warnell School of Forestry and Natural Resources, PO Drawer E, Aiken, SC 29802 USA; 3 Present Address: University of Nebraska, School of Natural Resources, Hardin Hall, 3310 Holdrege St., Lincoln, NE 68583-0961 USA; 4 Present Address: Trent University, Peterborough, Ontario K9L 0G2 Canada; 5 Present Address: Mountain Data Group, 115 N. College Ave. Suite 220, Fort Collins, CO 80524 USA

**Keywords:** Biological techniques, Genetic techniques, Population dynamics, Statistics

## Abstract

A critical element in effective wildlife management is monitoring the status of wildlife populations; however, resources to monitor wildlife populations are typically limited. We compared cost effectiveness of three common population estimation methods (i.e. non-invasive DNA sampling, camera sampling, and sampling from trapping) by applying them to wild pigs (*Sus scrofa*) across three habitats in South Carolina, U.S.A where they are invasive. We used mark-recapture analyses for fecal DNA sampling data, spatially-explicit capture-recapture analyses for camera sampling data, and a removal analysis for removal sampling from trap data. Density estimates were similar across methods. Camera sampling was the least expensive, but had large variances. Fecal DNA sampling was the most expensive, although this technique generally performed well. We examined how reductions in effort by method related to increases in relative bias or imprecision. For removal sampling, the largest cost savings while maintaining unbiased density estimates was from reducing the number of traps. For fecal DNA sampling, a reduction in effort only minimally reduced costs due to the need for increased lab replicates while maintaining high quality estimates. For camera sampling, effort could only be marginally reduced before inducing bias. We provide a decision tree for researchers to help make monitoring decisions.

## Introduction

Monitoring wildlife populations is essential for evaluating the success of management and conservation actions, developing management plans, and linking animal populations with their impacts on ecosystems^1^. Many monitoring techniques focus on estimating abundance or density of a population of interest^2,3^. Both abundance and density allow for evaluations of changes in populations over time or in response to management actions. Abundance estimates are useful to evaluate details about a specific population such as the viability of a population^4,5^, the effective population size^6^, determining hunting limits^7^, and to examine local impacts of changes in the system e.g.^8^. Density estimates are particularly useful for broader comparisons as they remove confounded issues with unequal areas e.g.^9,10^. Thus, when interested in comparing across habitats, study areas, study methods, and time, density is a more appropriate method of comparison^11,12^.

There are a range of abundance and density estimators that have been developed for wildlife populations (e.g., mark-recapture^13^, distance sampling^14^, removal sampling^15^). Different methods have their own strengths (e.g., robustness, ease of implementation, precision) and weaknesses (e.g., rigid assumptions, difficulty of implementation, variability)^16,17^. When the primary interest is in monitoring changes in populations issues with bias and precision are particularly concerning^18,19^. Biases in population estimators can lead to incorrect interpretations of natural systems or of management effectiveness^20^. If methodological biases are consistent (i.e. always positively or negatively biased to the same degree) the method would be useful for evaluating population changes but not useful in comparing across methods^21^. Issues with precision will impact the ability to determine the actual change in a system which may make it difficult to determine the best conservation or management action to take^18^.

Several studies have evaluated accuracy (level of bias) and precision (level of variability) of monitoring techniques^13,22–24^. In particular, mark-recapture estimates (with a mean maximum distance moved (MMDM) buffer to convert to density) were unbiased compared to truth but relatively imprecise, and performed poorly at very high or low densities^25^. Distance sampling methods were generally found to be unbiased but imprecise^25^. Spatially-explicit capture-recapture (SECR) methods generally perform better than simple capture-recapture methods, and capture-recapture methods are typically biased high compared to SECR estimates^26,27^. Non-invasive genetic mark-recapture methods were found to be biased high compared to camera trap mark-recapture methods as age differentials were not possible with the genetic methods^28^. Removal models have been shown to be biased when sample sizes are low^29^. Without considering costs, the optimal method may be dependent on unique conditions for a study system of interest. However, few studies have tried to examine the relationships between the cost of implementing specific techniques and metrics of estimator success^30^ making it challenging to select the optimal method that will achieve a desired objective under financial constraints^31^. Greater evaluation of methodology-cost relationships will facilitate selection of estimators that are sufficiently accurate and precise, while efficiently using financial resources.

Both invasive e.g., live-trapping and euthanasia^32,33^; and non-invasive e.g., genetic capture-mark-recapture^34–36^;, camera-trapping^37^; sampling methods provide data for density estimation. These field methods may have different baseline associated costs of implementation and differ in their robustness to change in sampling designs^38^. For some of these sampling methods, the cost of collecting additional data to improve accuracy and/or precision on their respective density estimation techniques may be relatively inexpensive compared with other sampling methods. However, the time spent processing the data (processing genetic samples or photo evaluation) may be time consuming. Therefore, evaluation is necessary to determine the relationship between cost of implementation of these sampling methods and the reliability of their density estimates, in terms of data gathered, precision, and accuracy.

Economic studies that compare the costs and benefits of information can directly estimate the value of information to a specific management goal^39,40^. In practice, managers may focus on a single biological metric e.g., observed species growth rates under managment strategies^41^, where resultant information can then serve as an evaluation tool for management actions. A certain management action may be deemed beneficial if information gained during management helps reduce bias in the biological metric of interest. Similarly, a decision maker who is risk averse may find greater uncertainty costly. For example, if the uncertainty around an abundance estimate is high and a different management action would be recommended if the abundance were at the high vs the low end of the confidence interval, a manager may want to invest more in getting a more precise estimate than risk making a poor management decision. Such individuals would value results with lower variances, and thus, be more willing to provide financial means for strategies that produce more precise outcomes. Tradeoffs in terms of costs and variance of results depend on the extent of risk aversion, which is typically modeled in a preference function mapping different outcomes into values. As we do not formalize preferences over species presence in such a way, references to risk aversion are subjective considerations.

*Sus scrofa* (known as wild boars, wild hogs, feral swine, or wild pigs) are considered a pest species across much of its range globally and cause damage to natural and anthropogenic ecosystems^42–45^. In the United States, wild pigs are an invasive species and cause tremendous amounts of economic damage to agriculture each year^46,47^. In addition, wild pigs have been linked to the extinction or extirpation of a number of native wildlife species globally^48^. We chose wild pigs as a study species to take advantage of ongoing research to evaluate density estimators for this species^29^. Knowledge of the cost effectiveness of density estimators for wild pigs will benefit management programs and allow informed decision making for optimized allocation of financial resources by agencies responsible for managing this invasive species.

In this study, we evaluate the costs of three field techniques (fecal DNA sampling, camera sampling, and removal sampling) commonly used to estimate population density of wildlife populations. We applied these techniques to a globally distributed and economically important invasive species, wild pigs (*Sus scrofa*), and evaluated how accuracy and precision of resulting density estimates change with sampling designs that reflect different amounts of financial resources. Further, we developed a decision tree to guide future researchers/managers in planning their density estimation studies.

## Results

During the fecal sampling, we collected 513 scats across all habitats: upland pine, bottomland hardwood, and mixed forest (Table 1). 176 scats contained sufficient DNA quality to pass the initial qPCR screening (i.e., amplified with a C_t_ ≤ 36). Of those, we were able to identify 159 samples to the individual level (bottomland = 33, mixed = 87, and upland = 36). From these samples, we detected 123 unique individuals across habitats. There were 19 individuals that had ‘recaptures’ meaning they were detected on subsequent days. A few samples were from the same individual but were detected multiple times on the same day and thus not counted as recaptures. During the camera sampling portion of the study, we captured 2,636 images in which wild pigs were present (Table 1). We identified 76 unique individuals (51 adults or subadults and 25 piglets) across the three study sites. A total of 59 wild pigs were caught in corral traps (32 adults or subadults and 27 piglets across habitats, Table 1). Using both the camera and removal sampling, we detected similar numbers of piglets, which varied considerably by habitat (e.g., for camera and removal sampling: 7 and 9 in the bottomland hardwood habitat, 0 and 1 in the mixed habitat, and 18 and 17 in the upland pine habitat, Table 1).

**Table 1:** Sample sizes of different sampling methods (fecal sampling, camera sampling, and removal sampling) by habitat for wild pig (*Sus scrofa*) population estimation at the Savannah River Site, South Carolina, in Feb-Mar, 2015.

		Bottomland hardwood	Mixed habitat	Upland pine	Total
Fecal sampling	Total # scats collected	276	174	63	513
	Total # scats not genotyped	243	87	27	357
	# unique individuals	31	59	33	123
	# of individuals redetected	2	14	3	19
Camera sampling	# of images with pigs	1637	376	623	2636
	# of Adults (Camera)	24	13	14	51
	# Piglets (Camera)	7	0	18	25
Removal sampling	# of Adults (Corral)	18	5	9	32
	# Piglets (Corral)	9	1	17	27

### Costs

**Figure 1:**
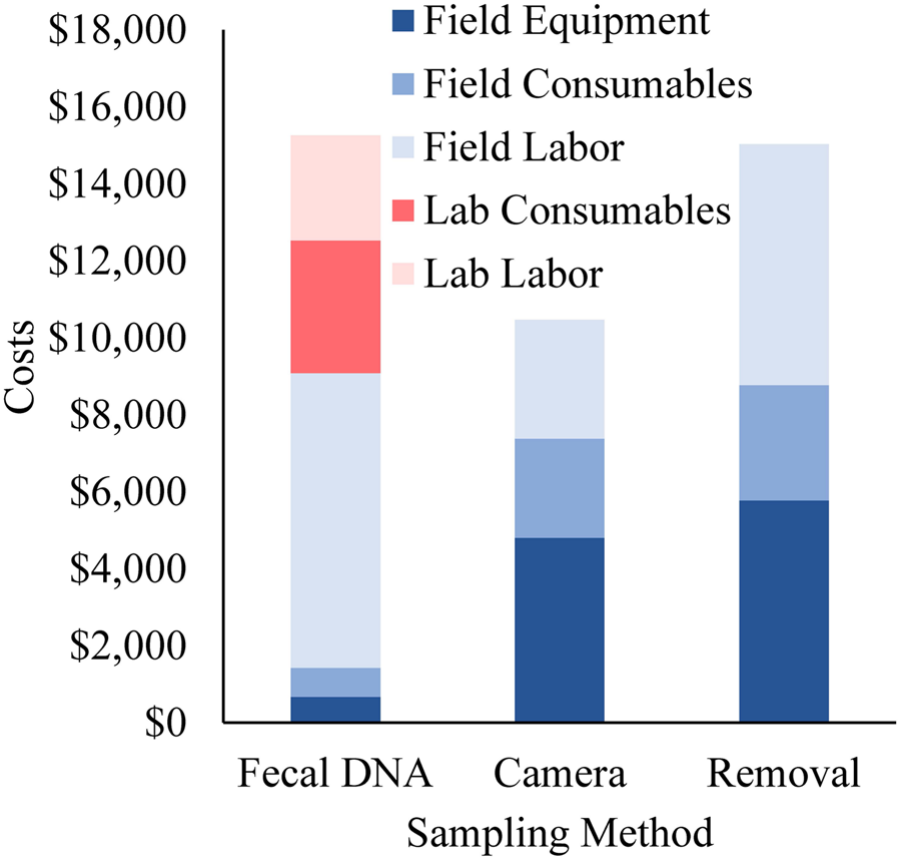
Costs of conducting density estimation on wild pigs (*Sus scrofa*) at the Savannah River Site, South Carolina in Feb-Mar 2015. Costs are shown by type (laboratory and field), category (equipment, consumables, and labor), and by sampling method (fecal DNA sampling, camera sampling, and removal sampling). There were no laboratory equipment costs.

Equipment costs were the largest portion of costs for both the camera and removal sampling methods (Fig. 1). However, equipment was the smallest proportion of costs for the fecal DNA sampling (~4% of total costs); this is largely due to the fact that studies rarely purchase laboratory equipment (e.g., PCR machines, extraction devices, etc.) for single genetic studies. Either they will have these on hand because it is standard genetic laboratory equipment or they send samples out to be analyzed by a genetics laboratory, as assumed in this study. Labor was the largest cost for the fecal DNA method (71% total, 44% from field labor and 27% from laboratory labor) and the second largest portion of costs for the camera and removal sampling methods (18% for cameras and 37% for removal sampling). Approximately 51% of the labor time for the camera sampling method was needed to examine and classify the camera data. Consumables were a smaller component of the fecal DNA, camera and removal sampling methods (average 18% of costs, Fig. 1).

The fecal DNA approach was the most expensive to implement (~$17,700), followed by the removal sampling approach (~$15,000), and then the camera sampling method (~$10,500). The fecal DNA method was the least expensive in terms of field costs (~$9,000), but laboratory costs added a substantial cost burden to this method. The fecal DNA method required the least financial contribution in terms of purchases (either consumables or equipment), however, it required a large investment of field labor. The camera and removal sampling methods were similarly balanced in terms of equipment costs and consumables costs, although the removal sampling required more labor than the camera method for the number of traps we used.

### Population estimates

**Figure 2:**
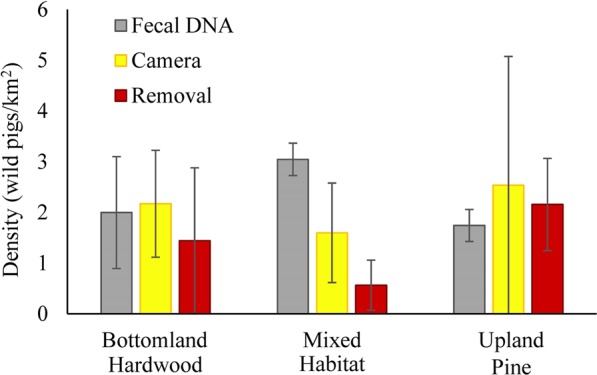
Density estimates of wild pigs (*Sus scrofa*) at the Savannah River Site, South Carolina, in 2015 by habitat from fecal DNA sampling (green), camera sampling (blue), and removal sampling (red). The 95% confidence intervals of density are shown for each estimate.

Spatially-explicit capture-recapture population estimates from the camera sampling data were given as densities as SECR models explicitly accounted for the spatial area impacted by the trapping grid design. The fecal DNA method and the removal model both estimate abundance. We used a mean maximum distance moved buffer around the fecal DNA transects and corral traps. Density estimates were similar across all methods in the bottomland hardwood and upland pine habitats (Fig. 2). The largest difference in the density estimates across methods was in the mixed habitat. The estimate from removal sampling was lower than the other estimates (Fig. 2). In contrast, the mixed habitat had nearly twice as many viable DNA samples collected than in the other two habitats (Table 1). Correspondingly, the estimate of density from the fecal DNA analysis was higher with lower variance than the other estimates for the mixed habitat as well as the estimated densities for the other habitats.

### Cost effectiveness

**Figure 3:**
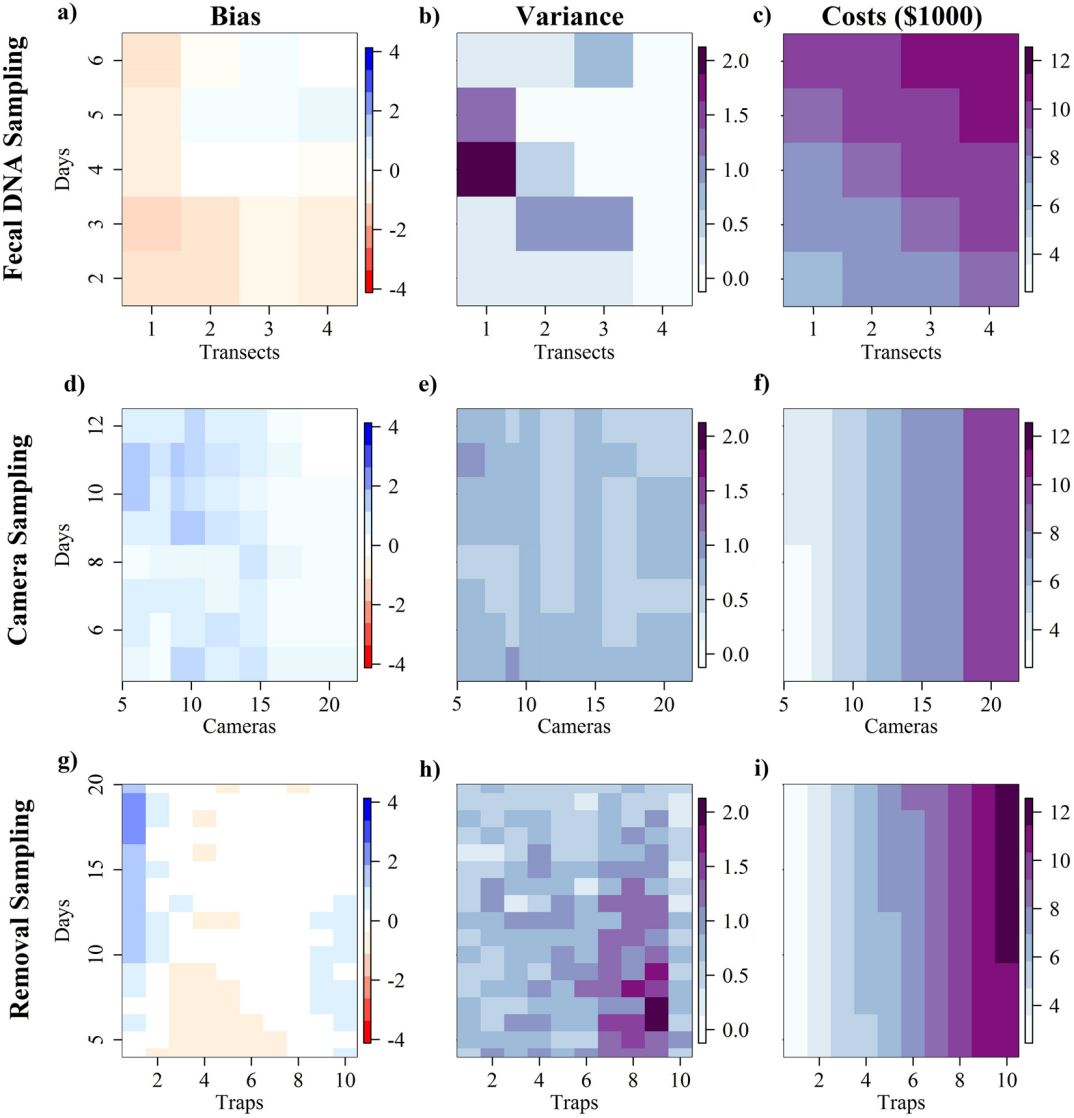
Impacts of reduction in effort on bias (difference in density from full data set model- positive biases are shown in shades of blue and negative biases are shown in shades of red; **a**,**d**,**g**), variance (larger values are shown in darker shades; **b**,**e**,**h**), and costs (costs are shown in $1000, darker shades represent more expensive methods; **c**,**f**,**i**) associated with density estimates of wild pigs from fecal DNA sampling (**a**–**c**), camera sampling (**d**–**f**), and removal sampling. (**g**–**i**) The effort reduction for the fecal DNA sampling is in terms of the number of transects and the number of days the transects are sampled. The camera sampling effort reduction is in terms of the number of cameras and the number of days sampled. The removal sampling effort is in terms of the number of traps and the number of days sampled. All estimates are based on wild pig (*Sus scrofa*) densities from Savannah River Site, South Carolina in Feb-Mar 2015.

The relative bias (within method difference from the full data model) for the fecal DNA sampling method was worse when there were fewer than four sampling occasions and when there were fewer than two transects (Fig. 3a). The estimates tended to be negatively biased as the effort was reduced (Fig. 3a). Based on the low number of recaptures, reducing the data further produced biased estimates. The precision was best (lowest variance) when all four transects were used (Fig. 3b). The reduction in costs was similar for reducing the number of transects and the number of sampling days (Fig. 3c). Costs ranged from a low of $6,384 for the least amount of effort (1 transect and 2 sampling occasions) to a high of $11,355 using all the effort (4 transects and 6 sampling occasions).

The relative bias for camera sampling was <1% only when all 20 cameras were used within the study area (Fig. 3d). When 20 cameras were used the relative bias was smaller when cameras were active for at least 11 days (Fig. 3d). Relative bias tended to be positive as effort was reduced for camera sampling data (Fig. 3d). An additional issue with the SECR approach is that models require a sufficient sample size (40+ detections based on our simulations) and spatial recaptures to be able to provide estimates (models fail when there is insufficient samples and spatial recaptures). When fewer than 12 cameras were used (regardless of the number of days) only 55% of replications had sufficient data to estimate density. Additionally, when fewer than 9 days of sampling was conducted, we needed to have 16 or more cameras to ensure all of the bootstrapped samples would have sufficient data to estimate density. By reducing sample size we also reduced the number of spatial recaptures to the lower threshold where SECR models begin to not converge. The variance around density estimates was not strongly related to sampling effort (Fig. 3e). Reducing the number of cameras had a substantially larger impact on the budget than reducing the number of days cameras were active (Fig. 3f), but that also increased bias. Costs ranged from $3,510 for 6 cameras and 4 days to $9,525 for 20 cameras and 12 days.

For removal sampling the results suggest that more than three traps are more likely to yield relatively unbiased estimates, particularly when trapping is conducted for 14 or more days (Fig. 3g). The estimates were relatively unbiased with 6 or more traps even with few days of trapping, however, the estimates were imprecise at fewer than 15 days of trapping (Fig. 3h). Costs increased more dramatically with an increase in the number of traps than with an increase in the number of a days a trap is active (Fig. 3i) (i.e. the cost of traps far outweighed that of labor). Costs range from a low of $2,947 with only one trap active for four days to a high of $11,795 for 10 traps active for 20 days. Our study used 10 traps and 14 days ($11,593), however, our simulation could test ranges of effort outside what we used.

### Decision tree

**Figure 4:**
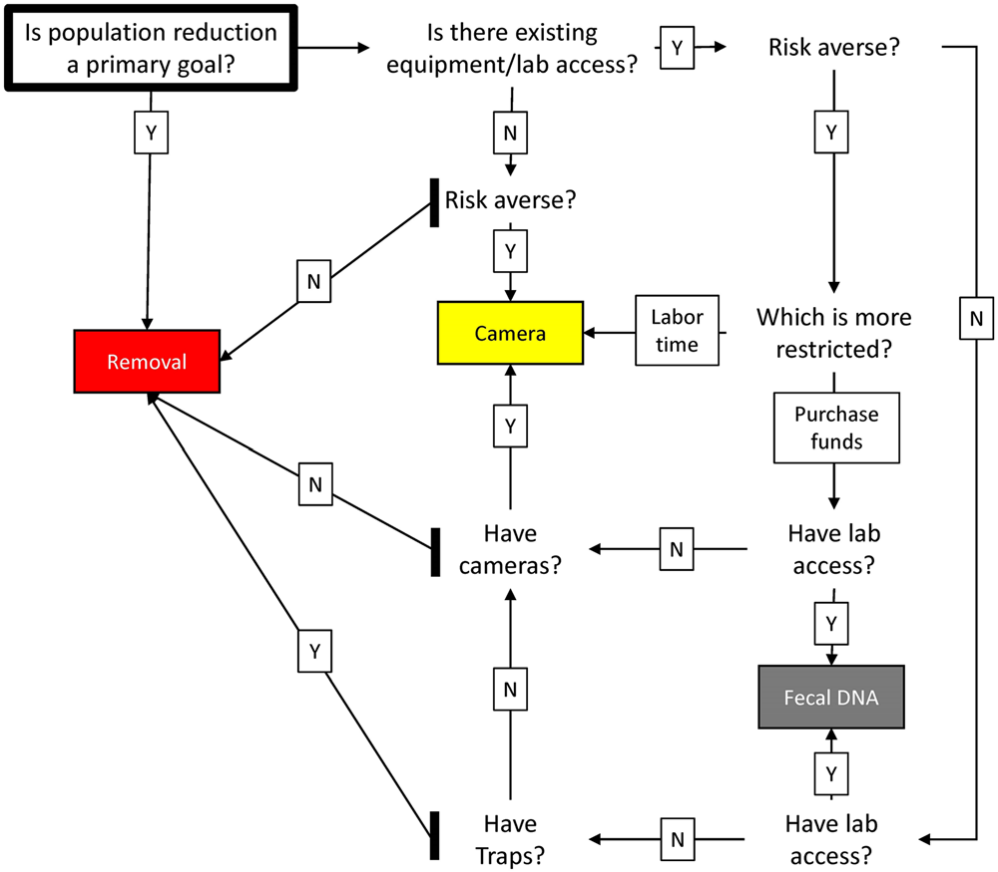
Decision tree comparing density estimation options for wild pigs (*Sus scrofa*) with less than 1% scaled bias based on field work on the Savannah River Site, South Carolina in Feb-Mar 2015. Selection of lowest cost outcomes for achieving less than 1% scaled bias by decision maker attributes: (i) Whether or not population reduction is a goal, (ii) existing equipment, (iii) preferences for smaller variance (greater degree of risk aversion), and (iv) if labor or equipment costs are more restrictive. If an objective is to use a non-invasive method then stop at the thick, vertical lines and proceed in the only option left.

We examined relative bias, variance, and costs with respect to all ranges of number of sampling units (i.e., transects, cameras, and traps) and days of sampling (e.g., 6, 12, and 20) for the three methods: fecal DNA sampling, camera sampling, and removal sampling. There were 3, 2, and 25 sample-unit/day combinations that satisfied the <1% scaled bias requirement for the three methods, respectively. When risk aversion was not a priority (larger variances are acceptable) we selected the method that minimized the overall costs. The sampling effort by method that minimized costs were for the fecal DNA sampling: 2 transects and 4 days, for the camera sampling: 20 cameras and 11 days, and for the removal sampling: 3 traps and 14 days. The methods that satisfied the risk averse condition were the ones that minimized the variance. The sampling effort by method that minimized variances were: for fecal DNA sampling 4 transects and 6 days, for camera sampling 20 cameras and 12 days, and for removal sampling it was 10 traps and 18 days. The resulting decision tree is shown in Fig. 4. As many factors will influence a decision tree (e.g., equipment on hand, cost of new equipment) the decision tree we have shown here may not suit all needs.

## Discussion

We examined three commonly applied wildlife monitoring techniques and evaluated their effectiveness (relative bias and precision) in relation to their financial costs of implementation when applied to management of invasive wild pigs. In general, estimates of density were similar across methods and similar to wild pig density estimates from other areas in the southeastern U.S.A. e.g., 1.07–2.74 wild pigs/km^2^ ^49^. However, each method had its own advantages and disadvantages in terms of implementation and cost effectiveness. The preferred method to estimate density in future studies will therefore be dependent on the unique conditions of that study system, which our decision tree can help give guidance on.

The fecal DNA field sampling required a large labor investment to establish transects, survey transects, and collect scat. Lab costs for similar studies will vary depending on: the access to lab resources, the number of fecal samples collected, and the number of PCR replicates that are done to overcome error rates^50,51^. In poor quality DNA, like that which frequently results from non-invasive fecal DNA sampling, it is common to produce 3–8 PCR replicates of genotypes in order to identify an individual with confidence i.e., multi-tube approach;^52^. Depending on the field versus laboratory costs, it may be more effective to take fewer samples in the field and run them more times in the lab than it is to put more effort in the field work to collect more samples that may not all be viable^53,54^. In our study only 20% of samples could be genotyped, but advances in the laboratory methods would potentially increase genotyping rates and thus reduce the need for greatly increased sample sizes. Additional cost savings in the lab would be to multi-plate the PRC replicates (i.e. run many samples simultaneously) which is standard practice in most labs and has the potential to significantly reduce lab labor costs.

The camera sampling method was the least expensive of the methods that we compared (given our sampling designs), with the primary cost being the purchase of camera equipment (Fig. 1). Cameras are cost effective for low-density species^31^ in part because the number of photographs to analyze are not excessive. Approximately 51% of labor costs for cameras was to examine photographs. These estimates are based on individual technicians examining every photograph. However, new technologies are being developed that have the potential to automate photo processing, which could greatly reduce this time investment^55^. Some studies have suggested camera sampling is a cost effective method for estimating population abundance or density^31,56^. However, Janečka *et al*.^28^ found camera trapping was almost twice as expensive in their study, but the cameras were active for over twice as long a time period than the fecal DNA sampling. We standardized our costs by using each method for a ~2-week period. In addition to the number of photographs (in part coming from the duration of the study) the time spent uniquely identifying individuals is also a function of the number of unique individuals, thus this method is more cost effective with lower density populations.

It is well known that removal estimators perform poorly when capture rates and/or densities are very low (and thus the number of animals captured is low; e.g., <10)^13,29,57^. Similarly, our removal sampling method only performed well when the number of animals captured was sufficient (>20 is desirable). The SECR method also failed when the sample sizes were too low, by not providing an estimate, which may be an advantageous feature because it allows the researcher to realize the limitations of the method. The relative estimates of density among the habitats were similar for removal sampling compared to camera sampling, although the removal sampling estimates were consistently lower than camera sampling estimates. This may suggest that the buffer used to convert abundance estimates from the removal data to densities is biased high. The buffer used was the mean maximum distance moved based on the camera data^16,29^. Although this buffer is commonly used, uncertainty in the buffer choice is rarely accounted for. Therefore, it is unknown if the contribution to the difference in density estimates with other methods is larger for the buffer or the removal estimate.

An assumption for each method is that there is demographic closure (i.e. no births, deaths, emigration, nor immigration). We limited the duration for each individual method to a small time frame (~2 weeks) to limit violations of the closure assumption. The total duration for all methods was ~1 month. If movement occurred on or off the study areas during the time frame this may contribute to differences in the estimates of density among the methods. Home range sizes within the Savannah River Site have been estimated to be 7.9 km^2^ for females and 14.0 km^2^ for males^58,59^. Generally, these home range sizes are smaller than our study areas and should have limited issues with immigration and emigration. If there were violations of closure, our estimates would be truly biased high (bias compared to truth) as we would be making inference to the population of individuals that used the study area, even briefly, during the study period and not just the population that was confined to the study area during the study period: i.e. a super population^60^. The largest issue with the closure would be with the fecal sampling method. If fecal samples occurred on the site from animals that simply moved through the study area prior to the study period this would result in estimates that are truly biased high compared to other methods, which tended to be the case in our study. Additionally, it was not possible to assess age with the fecal method, this also may lead to a positive bias in these estimates similar to Janečka *et al*.^28^ as the other methods in our study did not include juveniles in their density estimates.

Costs for the trap method were intermediate of the three methods we examined. The cost of trap equipment was the most expensive of the methods, and costs to set up traps, consistently check traps, euthanize trapped pigs, and reset the traps was also a substantial labor investment. Although the removal sampling method requires a significant financial investment, there is the added benefit of removing a pest species from the environment, which in some cases may be a primary objective. The costs reduced due to damage by wild pigs can be calculated as an offset to the out-of-pocket costs for the removal work when damage costs are available. The removal sampling method is advantageous as it can estimate abundance before and after a removal event as well as reducing the population simultaneously.

Our study was designed with multiple replicates of each sampling method which may not always be feasible due to logistical constraints. Therefore, we examined the impact on our density estimates if we had reduced our sampling effort. The sample sizes we used in this study are similar to those used in other studies^61–63^. For fecal DNA sampling, we examined the impacts of reducing the number of transects and the number of days transects were sampled. Reducing the number of transects or the number of days resulted in similar cost savings (~$710, ~6% cost reduction) because a similar amount of labor was required for each unit of these components. This is advantageous as a design can be tailored to account for either a space or time limitation without having much influence on costs (e.g., if only two transects will fit in a study area the number of days can be increased, or if the time available is restricted the number of transects can be increased and they will result in similar costs). However, since we had such low recapture rates we would caution against reducing effort.

When designing surveillance for a species with similar home range behavior as wild pigs, our results suggest at least 20 cameras (~1.3 cameras/km^2^) and 11 monitoring days should be used. Variances were large when fewer cameras were used, however it is important to note SECR models have greater imprecision than other methods because they explicitly take variation in animal movements into account where the other methods we examined do not. Reducing the sampling effort either in the number of days or number of cameras even by a small amount resulted insufficient data to estimate density using SECR models. Therefore, not much reduction in effort was able to yield similar density estimates to the full dataset, suggesting the effort we employed was close to the minimally sufficient sample sizes that should be used for camera studies to obtain consistent estimates. This type of analysis is not commonly done but can help researchers make informed design decisions going forward. In addition to needing a sufficient sample size we also have to ensure a sufficient number of spatial recaptures for SECR models to be fitted adequately^64^, therefore, spacing of the cameras needs to be appropriate for the species to ensure captures are possible on multiple cameras. With larger home-range sizes it may be necessary to increase the number of cameras per grid cell to account for a potential reduction in detection. The cost reductions for the camera sampling are primarily related to the number of cameras and reducing the number of days of sampling did not substantially reduce costs.

The removal sampling approach showed more of an interaction between number of traps and number of days reduced, and the relative bias and precision of resulting estimates. Costs were more strongly tied to the number of traps than the number of nights, as the cost for each trap is considerable and the total sampling time was relatively small (~2 weeks). More traps generally resulted in consistent estimates even at fewer days of trapping, however, these estimates were imprecise (Fig. 3). Removal estimators tend to be less biased and more precise in general than mark-recapture methods^65,66^. If the risk of having an imprecise estimate is not a strong concern than the cost of trap sampling can be greatly reduced by requiring only a third the number of traps (~1 trap per 6 km^2^, but trap for at least 10 days) and still have a consistent density estimate.

Costs estimated for traps in our study were for 5-panel, root-door corral traps^67^. Corral traps are preferable for estimating abundance through a removal estimator as they are likely to remove more individuals per trapping effort than box traps^68^ or snares^69^. The costs estimated per trap in our study (not including basic set-up equipment including t-post pounders, drills, hammer, etc.) was $550. However, costs per trap can range to several thousand dollars, so the cost-efficiency will depend on the choice of traps selected. We did not compare the efficacy of the different corral traps in this study, but there may be advantages to more expensive traps if they result in higher capture rates and thus more precise density estimates. It is also important to note we examined time frames for a short-term study (less than a month). If monitoring is to be continued, the proportional costs for increasing the time frame will become a larger contribution of overall costs than the upfront equipment costs that are generally a larger component in our study.

A large factor in the strengths and weaknesses in considering the three monitoring approaches we tested in this study would be the available resources. We showed the cost breakdowns for each density estimation method by equipment, consumables, and labor, as these resources may vary widely. In some situations there may be money for supplies, but not enough labor time based on current employee schedules, and thus camera sampling could be a preferable method. In other situations, there may be plenty of personnel time, but limited budget for expenses, therefore depending on available resources (traps, cameras, lab equipment) one method may be an obvious choice. To help guide selection of a monitoring technique that is appropriate to the available budget and monitoring objective, we developed a decision tree showing the minimally sufficient sampling effort required to gain reliable density information for the methods we used (Fig. 4). This type of tool is particularly useful for managers or researchers attempting to monitor wild pigs. However, results of our study are based on one study in South Carolina. The decision tree may change under different conditions or species based on changes in movement behavior, capture rates, identifiability, or genotyping rates. As with any research study it is important to consider the unique aspects of a system before beginning.

Monitoring wildlife populations is a constant challenge and is often conducted with limited resources. There are many methods to estimate wildlife population density and the optimal method will depend on many factors: the species of interest, the habitat, the seasonality, the resources available, etc. As, different types of estimation methods may vary in cost effectiveness in different conditions, comparing cost effectiveness of estimators under different conditions helps to understand how bias and precision can be minimized for a particular application and budget. Future work may want to expand to other estimation methods and to compare the efficacy of different traps or cameras as used in this study.

## Methods

### Study area

The Savannah River Site (SRS) is a 78,000 ha U.S. Department of Energy facility in South Carolina near the border with Georgia in the southeastern United States. The primary habitats of SRS were upland pine (~68% of the SRS) and bottomland hardwood forests ~22% of the SRS; described in^70^. We selected a study site in each of these forest types and in an additional site, hereafter mixed habitat, which consisted of upland pine forest containing riparian areas. Elevation of the SRS ranges from 30–115 m above sea level. Climatic conditions of the SRS are described in Kierepka *et al*.^71^ and are generally characterized by warm temperatures and high humidity. Wild pigs have been lethally controlled at the SRS since 1952 in an effort to mitigate damage^72^. Two weeks prior to the beginning of this research, our study sites and a 2 km buffer surrounding those areas were closed to contractors, preventing trapping or hunting during this project. In addition, roads surrounding and throughout study sites were monitored for wild pig mortalities due to vehicle collisions two weeks prior to and during the study. Given the short time frame of our sampling methods we assumed no births, nor immigration or emigration on or off our study areas.

### Field methods

All field methods were carried out in accordance with approved guidelines and research protocols (University of Georgia IACUC permit A2015 05–004-Y). Within each study site (i.e., bottomland, mixed, upland), we applied three common field techniques to gather data February to March in 2015. These techniques were applied within one month in the following order: (1) fecal sampling for non-invasive DNA mark-recapture (fecal DNA sampling), (2) using photographs from camera traps to identify individual animals based upon natural marks (camera sampling), and (3) live-trapping and lethal removal (removal sampling).

**Figure 5:**
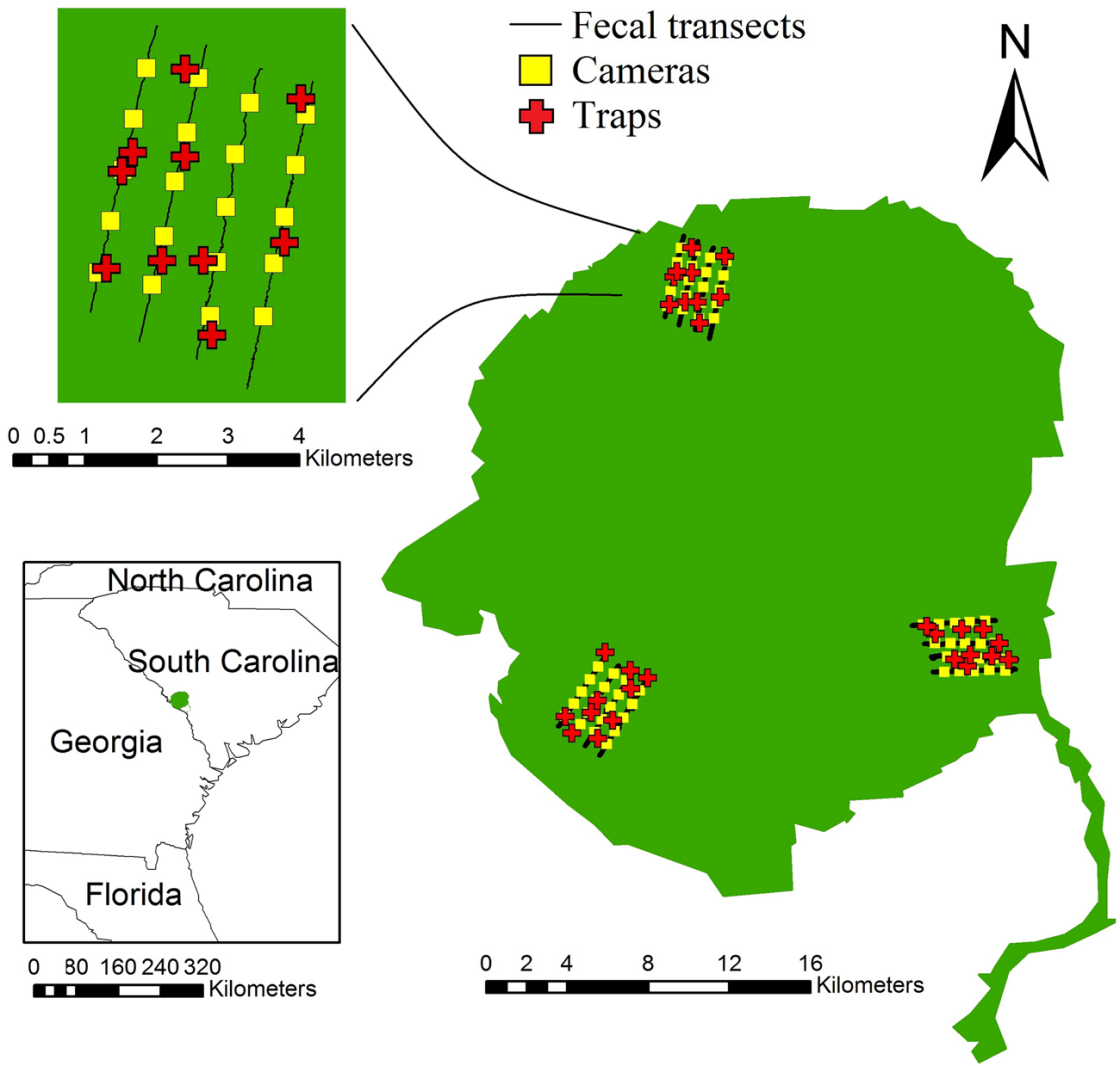
Map of the Savannah River Site, South Carolina with three study sites where wild pig (*Sus scrofa*) monitoring was conducted in Feb-Mar 2015. A detailed look at the northern site shows locations of the fecal sampling transects (black lines), overlayed with the camera sampling locations (yellow squares), and the trapping locations (red crosses). The trapping locations were selected to maximize wild pig detections within randomly selected 1 km^2^ grids.

In each study site, we established four fecal DNA sampling transects, each 4 km in length. Transects were parallel and spaced approximately 750 m apart (Fig. 5). We expected recaptures would be possible among transects within a study site (although no recaptures were detected across study sites). One person walked a single transect on each sampling occasion. We collected fecal samples along transects every other day for 12 days for a total of 6 sampling occasions per study site. Research by Kierepka *et al*.^71^ demonstrated that accurate genotypes could be attained from wild pig fecal samples with five days of environmental exposure, excluding rain; therefore, our sampling scheme should be minimally affected by exposure issues. As in Ebert *et al*.^62^, we employed a modified adaptive cluster sampling protocol, which is a method designed to help detection of rare but clustered samples, described in^73^ to maximize collection of fecal samples and account for social behavior of wild pigs. From each scat we encountered, we collected ~0.5 g of scat from the outside of a fecal deposit, and stored the sample in a 1.5 mL Eppendorf tube filled with molecular-grade 90% ethanol. We recorded the GPS point for each fecal sample collected. We stored fecal samples in a −80 °C freezer until DNA extraction. Smaller scat were harder to detect (Keiter *et al*. 2016) and less likely to be viable for typing information, limiting the inclusion of piglets in the genetic analysis. Since piglet movement is not independent of sow movement and birth pulse timing will heavily influence detection rates by habitat, we limited our analysis to adult and subadult wild pigs in the camera and removal sampling portions of the analysis.

Once the fecal DNA sampling was completed, we initiated camera sampling in the same study areas within two days. We established a 5 × 4 grid of white-flash trail cameras (Scoutguard SG565FV, HCO Outdoor Products, Norcross, USA; Fig. 5). We placed cameras along the fecal sampling transects 750 m apart (±75 m) in locations that would maximize the probability of animal detections based upon local habitat conditions or evidence of pig presence (e.g. rooting, scat, etc.), and baited them with 22 kg of whole corn placed on the ground in front of the camera. Cameras had motion triggers, with a 3-minute delay between trigger activation, and were programed to take 3 pictures, 5 seconds apart, when triggered. Camera traps were active for 12 days and rebaited midway through. We identified individual animals using natural marks (e.g., pelage, scars, size, sex) and associations with other individuals from camera photos, as in Sweitzer *et al*.^74^. Wild pigs in our study area are largely spotted due to the prevalence of domestic pig ancestry and may be more easily identifiable to the individual than some other wild populations of pigs^29^. Similar to Sweitzer *et al*.^74^, we believe that we were able to accurately identify untagged individual pigs within our population, and assume that our identifications were accurate for purposes of our analyses. The approximate age and sex of each individual also was determined. We defined piglets as weighing approximately 20 kg or less^75^. To create individual capture histories, each 24-hour period that a camera was active was defined as a capture occasion. Following camera sampling, we placed 10 corral traps in a grid of 1 km^2^ cells in each study site in areas with recent pig activity or in what was judged to be the best habitat if no fresh activity was found (Fig. 5). We pre-baited traps with whole corn for three days, and live-trapping occurred for 14 days in each study site (i.e. 140 trap-days per habitat type). Captured pigs were euthanized by cranial gunshot (University of Georgia IACUC permit A2015 05-004-Y).

### Laboratory methods

Full details of laboratory methods can be found in Kierepka *et al*.^71^. To conduct capture-recapture (CR) analyses on the genetic samples, we had to first produce microsatellite genotypes for individual identification from each fecal sample. Fecal samples are known to provide degraded DNA^62^. To limit processing of samples that would not produce complete genotypes, we pre-screened the samples via quantitative polymerase chain reaction (qPCR). We performed two DNA extractions from each fecal sample and ran qPCR analysis targeting the smooth muscle protein 22-alpha gene TAGLNsus;^62,71^ on each extract. Amplification conditions for qPCR followed Kierepka *et al*.^71^. Samples that amplified with a C_t_ of ≤36 cycles for both of the two extractions were further analyzed for individual identification^71^.

Since pigs travel in social groups, the genetic dataset needed to be able to differentiate between close relatives. We identified 9 microsatellite loci FH1589, FH2148, Sw911, Sw936, Sw2021, UMNp358, UMNp445, Susc11, and Susc27^76–78^; that had sufficient power to differentiate relatives and had high amplification success in samples with high degradation common in fecal samples. Each DNA extract was amplified at least 3 separate times with positive and negative controls to minimize errors i.e., allelic dropout and false alleles; see^71^. We utilized methods described in Kierepka *et al*.^71^ for amplification of microsatellite loci and analysis. Individuals genotyped at >7 loci obtained a probability of siblings (P_SIB_ < 0.0007), we excluded samples with less than 7 loci. For each locus we ran three PCRs per sample. All unique genotypes were entered into the program Genecap 1.4^79^, a Microsoft Excel extension that identifies individuals with matching genotype as well as those with one and two allele differences (i.e., cases of probable allelic dropout or closely related individuals).

Given DNA degradation in fecal samples, we expected some level of allelic dropout within our dataset^71^. Therefore, we focused on those with 1-allele differences as possible recaptures. Individual samples with 1-allele differences were considered putative matches under the following condition: the mismatch had to be at a locus where a heterozygote occurred in at least one of the replicate runs. These samples were then re-run a final time with increased amounts of template DNA to verify a case of allelic dropout. If the resultant genotype was still a mismatch, then we considered the samples to be different individuals.

### Cost data

We recorded the cost (USD) of implementing each sampling method during field and laboratory work. We broke the costs into two major types: field and laboratory costs, as managers are more likely to use their own staff and equipment for the field components and are more likely to send genetic samples to a laboratory for genotyping and analyses. Within those broad types, we split costs into three categories: equipment, consumables, and labor. Field equipment costs included items such as camera equipment (e.g., cameras and memory cards), trap components (e.g. panels and gates), tools (e.g., drills, bolt cutters, T-post pounders), handheld GPS units, and rifles to euthanize captured pigs. We used Scoutguard white flash cameras ($159 each) which are a lower to mid-range priced trail camera. We used two types of corral traps: a root gate with standard corral panels (~$550 per trap) and a JagerPro MINE corral trap (~$1,860 per trap). Consumable goods included fuel, bolts, wire, ammunition, gloves, and fecal DNA sampling material (e.g., ethanol, tubes, labels, tweezers). We calculated labor costs based on the number of person-hours required to implement each of the different field methods, including driving times to and from field sites and the frequency of visits required. For fecal DNA sampling there was both field and laboratory labor. The field labor costs for fecal DNA sampling included the person-hours necessary to walk transects and collect scats every 48 hours and the laboratory labor time included the laboratory technician time. For the camera sampling field method, the labor category included the time required to initially place and bait, re-bait, and collect camera traps, in addition to the time necessary to process and analyze camera images, identify individuals, and to create capture histories. Labor for removal sampling included the time spent evaluating the habitat to effectively place traps, transporting trap materials prior to building, constructing and de-constructing corral traps, checking trap-lines, and euthanizing captured animals.

We split laboratory costs into the same categories. We assumed there was no lab equipment costs as the lab used for the fecal DNA work already had all necessary equipment. The consumables include consumable plastics and extraction kits used per sample, the reagents for the qPCR screening and microsatellite panel amplification PCR, as well as instrument usage costs per sample. The labor costs were recorded explicitly for this study; however, labor costs are highly dependent on the lab instrumentation available (automated extraction machines, number of thermocyclers, throughput capability, etc.) and technician experience. Our estimates of lab costs are comparable to commercial lab prices based on the scale of this study, however, with larger studies, commercial labs may offer discounts as economies of scale will prevail over throughput capabilities of independent labs. Therefore, we estimated a range of times that would be required by a dedicated lab technician based on our lab instrumentation and technical expertise.

### Population estimators

Our study was conducted in a relatively short time period (~1 month) to try and ensure demographic and geographic closure existed among adult animals in each study site. The most common sources of mortality for adult wild pigs in this area are human harvest and vehicle collisions^80^. No harvesting nor culling (besides our removal efforts) nor vehicle deaths occurred within ~2 km of the study sites during this project. Typical gestation period for wild pigs is 115 days^81^ thus we are limiting the probability of births during our study by having a 1 month study period. Average home range sizes for wild pigs are variable, ranging from 0.3 km^2^ to 48 km^2^ with males generally having a larger home range e.g.^63,72,82^. Estimates of home range sizes in South Carolina are considerably smaller, 1.9 km^2^ to 14 km^2^ ^59,72,83^. Therefore, we think it is reasonable to assume there was neither immigration nor emigration during our study period.

We analyzed genotypes produced from fecal DNA sampling using closed population abundance models^84^. These methods estimate abundance (N) while accounting for imperfect detection and allow for initial detection (p) and redetection (c) rates to vary. In the case of genetic mark-recapture from fecal DNA sampling^85^, once an individual genotype was identified from its scat, it was determined to be ‘marked’ (in our case ‘detected’). ‘Recaptures’ (in our case ‘redetections’) occurred when scat from a previously genotyped individual was detected on a subsequent day. The data from all three habitats were analyzed jointly, which allowed us to compare the detection and redetection rates across habitats while estimating a separate abundance for each habitat. We assumed the study areas were spatially independent because distances between each area (>13 km) was far greater than average home range sizes of animals on the SRS^59^. We fit a model in which detection rates were different than redetection rates and detection rates varied by habitat.

We analyzed the camera sampling data using spatially-explicit capture-recapture (SECR) analysis implemented in package ‘secr’ in program R^86,87^. Our *a priori* model included constant density (D), movement varying by habitat (σ), and the probability of detection varying by behavior and time (g0).

For the removal sampling data, we used a removal modeling framework implemented in a Bayesian hierarchical approach^15,88^. Removal models jointly estimate initial abundance and capture rate by assuming a constant capture rate and evaluating the ratio of animals removed on subsequent removal events to estimate the proportion of the population removed (capture rate) and back calculate to estimate initial abundance^15^. Our adaptation of this method accounts for variations in sampling effort in this case the number of active traps^32^. We implemented the model coded in R as in Davis *et al*.^32^.

Spatially-explicit capture-recapture population estimates from the camera sampling data were estimated as densities because SECR models explicitly account for the spatial area impacted by the trapping grid design. The fecal DNA method and the removal model from corral trapping both estimate abundance. Therefore, the abundance estimates were converted to density to make estimates comparable. We used a buffer around the fecal DNA transects and corral trap grid equal to the mean maximum distance moved (1.123 km) based on results from Ivan *et al*.^16^, Gerber and Parmenter^22^, and Keiter *et al*.^29^.

### Cost effectiveness given effort

To determine cost effectiveness functions of different density estimators, we examined the relationships between costs (USD), bias (i.e., within method difference from density estimate with full data), and precision (i.e., variance of the density estimate) within a sampling method as we systematically reduced effort (i.e. number of days or number of transects/cameras/traps used). Thus, the cost effectiveness was calculated as dollars spent per unit of bias or precision of the density estimator. For each method (fecal DNA sampling, camera sampling, and removal sampling), we used a bootstrapping approach to sample from the data as if we had collected fewer samples to determine how smaller sample sizes would have resulted in different levels of bias and precision. We then compared the bias and precision of the estimated densities with the amount of cost savings that resulted from the reduced sampling regime. We compared the costs for the less expensive corral trap for the bootstrapping approach. Since we did not know the true density of wild pigs in our study, we were not able to obtain the actual bias in our estimates as a function of reduced effort. We assume that the model that uses all of the data is likely to be less biased than estimates based on a reduced dataset. Thus, we studied the difference in the estimates for the reduced datasets relative to the full data, which we term ‘relative bias’. We also compared how the variance associated with density estimates changed with respect to variations in effort (and thus costs).

For fecal DNA sampling, the costs are related to the field component of walking transects and collecting feces, as well as the number of samples collected at each sampling occasion which influence the amount of laboratory work associated with genotyping each sample. The number of samples collected varied by transect and sampling occasion. We used the average sample size by transect and occasion to estimate costs associated with a given sampling event.

For camera sampling, we reduced each of the number of camera traps and the number of days cameras were active. SECR models require a sampling design with camera spacing that is strategically related to the animal’s movement (i.e., spacing that is able to capture individual-level space use, in our case a 750-m radius). We also maintained a rectangular shape as we were interested in the effects of reducing camera number and not the optimal shape of the design of a camera array. We reduced the number of cameras used in the analysis by iterating through all combinations of rectangular grids that could fit in the full 4 × 5 grid array. This resulted in 47 different camera configurations. We recognized that in smaller sampling designs there would not be additional bait piles on the landscape as there are in our study (since we are only theoretically removing cameras). This is likely to impact how animal movement would change based on the study design, but should not bias the estimate of density. We also tested the relative impact of reducing the number of days cameras were active compared to reducing the number of cameras used.

For removal sampling (as with the other methods), we assumed the population of interest was closed to demographic changes during the sampling period. Based on this assumption the only changes in the population were due to our removal efforts. It was not possible to use a similar bootstrapping approach for reducing the number of traps in the removal method because dropping data from a trap that had removed individuals would violate the closure assumption. Therefore, to examine effects of reducing the number of traps on abundance estimates with the removal model, we used a simulation approach. We generated animal home range centroids on a landscape using a partial Poisson clustering algorithm^89^ to mimic the gregarious nature of the species. The density of animals generated was based on the mean estimated density across all methods. We placed 10 traps on the landscape using the same spatially balanced design (one sample per spatially discrete grid cells) as was used for the field work. We assumed the probability of an animal encountering a trap would decline as the distance from the animal’s home range centroid to the trap increased. We modeled this as a truncated Gaussian relationship^90^. The standard error of this Gaussian relationship was set to the estimated movement parameter (σ) from the spatially-explicit capture-recapture analysis. The capture rate, given an animal encountered the trap, was set so the simulated detections resembled observed capture patterns. Using the simulated data, we compared the effects of reducing the number of traps and reducing the number of trap days on the true bias from simulated data.

Because the removal models estimated abundance instead of density, we also needed to determine the area impacted by traps for the conversion of abundance to density. We hypothesized that the area impacted by traps would relate to both the number of traps and the number of trap days. Using the true density from simulations, we calculated the area impacted by the trapping effort by dividing the abundance estimate by the true density. We then used a linear model to estimate the relationship between buffer size and the number of days and number of traps. Bias in density estimates was calculated as the difference between the known true density and the estimated density (using area calculated from the appropriate buffer).

### Decision tree

We used the cost/benefit data to create a decision tree to guide future research and management with these methods. We started with the assumption that we would not want to choose a method that with high bias, thus we limited our options to those that had an absolute scaled bias (the absolute difference in the estimated density with the reduced sampling effort compared to the density with the full sampling effort, scaled by the density estimate of the full data set) of less than 1%. Of the options for each method that satisfy the bias criteria we calculated the overall costs, the labor costs, the laboratory costs, and the variance to highlight the strengths of different methods. In general, the optimal decision will be the one that minimizes costs while satisfying the research or management priorities. One priority may be increased precision, as a decision maker who is averse to uncertainty may find methods producing estimates with greater variance costly in the sense that imprecise methods behave differently due to the possibility of varied outcomes. Thus, more risk averse decision makers value lower variance in that they would be willing to pay for a more certain outcome as opposed to facing a range of varied ones. Some decision makers may be more restricted in terms of labor time available than funds available to purchase equipment and thus we incorporate that priority in the decision tree. Finally, the costs will depend on equipment on hand and lab accessibility, so we included those options in the tree.

## Data Availability

Data will be available on Dryad upon acceptance.
